# Alcohol consumption pattern in western Nepal: findings from the COBIN baseline survey

**DOI:** 10.1186/s12888-019-2264-7

**Published:** 2019-09-12

**Authors:** Tara Ballav Adhikari, Anupa Rijal, Per Kallestrup, Dinesh Neupane

**Affiliations:** 1 Nepal Development Society, Bharatpur, Chitwan Nepal; 2 Department of Public Health, Aarhus University, Aarhus, Denmark; 3 Department of Regional Health Research, University of Southern Denmark, Odense, Denmark; 4 Welch Center for Prevention, Epidemiology and Clinical Research, Johns Hopkins Bloomberg School of Public Health, Baltimore, USA

**Keywords:** Alcohol consumption, Alcohol drinking, Bing drinking, Nepal, Gender difference

## Abstract

**Background:**

Harmful use of alcohol is a global public health problem. Differences in alcohol consumption patterns may add valuable information to the design of public health interventions to prevent excessive use of alcohol, which is yet missing in Nepal. Hence, the purpose of the study is to determine the prevalence, patterns of alcohol consumption and socio-economic correlates of lifetime alcohol consumption and binge drinking in the semi-urban area of Pokhara Municipality.

**Methods:**

The cross-sectional data used in this study were collected as part of the COBIN study to understand alcohol consumption patterns and frequency and to determine correlates of lifetime alcohol consumption and binge drinking in the semi-urban area of Pokhara Municipality, Nepal.

**Results:**

Out of 2815 study participants, 35.6% had ever used alcohol in their lifetime (Male 67.2% and Female 18.9%). Among 571 respondents who drank alcohol within the past 30 days, 77.1% male, and 46.9% female reported binge drinking behaviour. On average, males consumed 8.8 ± 0.3 standard alcohol drinks on one occasion, while females consumed only 4.4 ± 0.3 alcoholic drinks. Male (OR = 16; 95% CI: 12.1–21.1), older adults (OR = 1.5; 95% CI: 1.2–1.7) and people belonging to disadvantaged ethnic group (OR = 6.1; 95% CI: 4.9–6.3) had higher odds of lifetime alcohol consumption than their respective counterparts. Whereas, male (OR = 7.9; 95% CI: 4.3–14.6), having higher educational status and agriculture as the occupation had higher odds of binge drinking.

**Conclusion:**

Alcohol consumption frequency was significantly higher among males than females in Western Nepal. Although national program and policies should recommend reducing alcohol consumption in general, targeted interventions are needed for males aged 45–65 years of age and certain ethnic groups (Dalit and Janajati).

## Background

Globally, harmful use of alcohol is estimated to result in 2.8 million deaths accounting for 6.8% of all deaths among males and 2.2% of all deaths among females in 2016 [1]. It is also considered as the 7th leading risk factor for both mortality and morbidity [1]. The effects of continued excessive use of alcohol- such as liver cirrhosis, cancer, and alcohol-induced road traffic accidents and injuries have detrimental consequences to the physical, mental, social, and economic wellbeing of the individual and constitute a burden to the already weakened health system of the country. A systematic analysis of the Global Burden of Disease 2016 suggested that no amount of alcohol consumption is safe [2]. Moreover, it further halts the achievement of Goal 3 of Sustainable Development Goals (SDGs) [3] focusing on ensuring good health and wellbeing at all ages by 2030, which significantly strides for good mental health and denotes the strengthening, prevention and treatment of the harmful use of alcohol [4].

The amount of alcohol consumed, and patterns of drinking play a significant role in developing alcohol-related harm. Heavy episodic alcohol drinking (HED) or “binge drinking” (five or more standard drinks for males and four or more standard drinks for females on a single occasion) has more adverse health consequences than non-HED [5]. The standard drink refers to 14 g of pure alcohol [6]. HED has been associated with cognitive impairments such as frontal lobe and working memory deficits [7, 8], and produce long-term disabilities among alcohol withdrawals [9].

Alcohol use is the eleventh most leading risk factor for Disability Adjusted Life Years (DALYs) in Nepal [10]. According to the WHO STEPS survey 2013 in Nepal 17% (male 28%, female 7.1%) of the surveyed population had consumed alcohol within the last 30 days; of these, 18.6% male and 2.9% female were binge drinking [10]. Alcohol use in Nepal is socially and culturally accepted in many ethnic groups, and the consumption has been increasing over the years across all ethnicities and age groups [11]. Although Nepal still has a high alcohol abstinence rate, those who drink consume almost five times more alcohol compared to the global average (28.8 vs 6.2 l of pure alcohol) [12].

So far, only few studies have investigated alcohol consumption patterns and frequency in Nepal. A cross-sectional study by Thapa et al. among squatters in Kathmandu valley found that the consumption of alcohol among urban poor was higher than the national average and showed gender differences in drinking pattern with males more likely to consume alcohol as compared to females [13]. However, the sampling in the study was purposive in nature hence limiting comparability. Likewise, further analysis of WHO STEPS survey Nepal 2013 showed that females from upper age groups, hills, and with no formal education were found likely to be consuming more alcohol than their other female counterparts [14]. Thus, a community-based study with a comprehensive gender-based assessment of alcohol consumption, frequency and pattern is still lacking in Nepal.

On February 2017, the Government of Nepal passed the National Policy on Regulation and Control of Alcohol to ensure high-quality life of every citizen by reducing the detrimental health and social consequences of the harmful use of alcohol. This policy envisions for a separate mechanism from local to central level to monitor implementation. Similarly, the Ministry of Health and Population is currently working to restructure health governance, draft a new health policy and design a province-based health care delivery system for the new federal structure of the country. In this light, the study on alcohol consumption at the local level can provide relevant information for tailored alcohol policy.

This study is derived from a survey of the community-based management of non-communicable diseases (COBIN) from western Nepal. The COBIN study aimed to determine the effect of home-based health education and screening of blood pressure in adults by female community health volunteers (FCHVs) [15, 16]. Moreover, the COBIN study also estimated the prevalence and risk factors of hypertension, such as alcohol consumption. Thus, based on data from COBIN the purpose of the current study is to determine the prevalence, patterns of alcohol consumption and socio-economic correlates of lifetime alcohol consumption and binge drinking in the semi-urban area of Pokhara Municipality of Nepal.

## Methods

Data for the current study were collected as part of the Community Based Management of Hypertension in Nepal (COBIN) study, initiated in the semi-urban area of Pokhara Municipality in 2013.

### Study area

The study area is a semi-urban area of Pokhara Municipality (formerly known as Lekhnath Municipality) in Kaski district of Gandaki Province, of western Nepal. The adult (> = 18 years of age) population of the municipality according to the database of Election Commission of Nepal in 2007 was 39,511 persons living in 9500 households.

### Study population

People 25–65 years of age who were listed in the voter list of 2007 of a semi-urban area of Pokhara Municipality, Kaski district were eligible for inclusion in the baseline survey. People who were under 25 years of age, severely ill, pregnant women, who were unlikely to be in the community throughout the intervention period and those who declined to give consent were excluded from the study.

### Sample size and sampling procedure

The sample size was calculated with a 95% CI (z = 1.96) and a 5% margin of error. As the primary study objective of the COBIN was to estimate the prevalence of hypertension and its risk factors, the prevalence of hypertension was taken for estimating the sample size for the study. The prevalence of hypertension considered for the study was 25% from the recent WHO STEPS survey of Nepal with the design effect of 1 (for random sampling) and a response rate of 80% in accordance with the guideline. Based on an age and sex estimate, eight strata were identified, i.e. four age groups for each sex (male and female). The total sample size calculated was 2882. Of which 2% of the sampled population either did not give consent or were not available during the household visits. Thus, data from 2815 participants were included in this study. The sample size calculation for the COBIN trial has been discussed in detail previously [15, 16].

### Study design

The study is cross-sectional in nature. Cross-sectional data were collected among the 2815 respondents of the study area.

### Study tools

The WHO STEPwise approach to non-communicable disease (NCD) risk factor surveillance tool was used for data collection. This tool has previously been validated and used by Nepal Health Research Council in a Nepalese context [17]. The WHO STEPwise questionnaire includes physical measurements (height, weight), sociodemographic information (age, sex, family size, occupation, income, education, etc.), lifestyle factors (salt consumption, smoking, alcohol, physical activity, etc.), and blood pressure measurement. The current study only uses data on sociodemographics and information on alcohol consumption. For alcohol consumption data, a showcard was prepared, including bottles and glasses which can be used for drinking purpose. The card was shown to the participants, and they were asked to estimate the alcohol intake for the last 1 week (on a daily basis). The outcomes and exposures were measured as described below:

### Outcome measurements

The outcome was measured as follows:

- Lifetime abstainer: Those who had never consumed alcohol in their lifetime
- Former drinker: Those who used to drink alcohol but have abstained for the last 12 months
- Current drinker: Those who had consumed alcohol at least once during the last 12 months.
- Binge drinker: Excessive alcohol consumption on one occasion (or within a 2 hours), consisting of five or more standard drinks for males and four or more standard drinks for females during the past 30 days [18].
- Alcohol use in the past 7 days: Consumption of standard drinks in the past 7 days in a week (Sunday to Saturday)

### Exposure measures

The age of the person was grouped into two categories: 25–44 years and 45–65 years. Educational level was grouped into two categories: High education (completed secondary education and above) and low education (completed primary education and below). Occupation of the person was categorized into agriculture, labour homemaker, employee, unemployed and others. Ethnicity was coded in accordance with Health Management Information System, Department of Health Services, Ministry of Health and Population [19], however, for analysis, it was further grouped into advantaged ethnicity (Brahmin/Chhetri) and disadvantaged ethnicity (Dalit, Janajati and others).

### Data analysis

Univariate, bivariate, and multivariable analyses were conducted in STATA version 14. The methods adopted in a sample analysis comprised descriptive statistics on alcohol consumption by gender. Gender-wise percentage and frequency distribution of sociodemographic characteristics (age, ethnicity, marital status, education, occupational status) and alcohol consumption patterns were calculated. The association between alcohol consumption and sociodemographic characteristics was assessed in bivariate analysis through Chi-square test. These sociodemographic variables were considered in multivariable analysis to compute the adjusted odds ratio. We also checked if gender interacted with other exposure variables. However, no interaction existed between other exposure variables hence, was not reported in the study. Thus, we controlled each of these variables in our adjusted model.

## Results

### Sociodemographic characteristics

Of the total of 2815 participants, the majority were female (65.5%), married (89.5%), had secondary or higher-level education (52.1%) and belonged to the 45–65 years age group (54.0%) with a mean age of 45.2 ± 10.2 years.

### Alcohol consumption pattern and behaviour

Table 1 shows the difference in alcohol consumption pattern and behaviour among male and female respondents in the study and these results were found to be statistically significant at 95% CI. Among 2815 respondents, 64.4% had never consumed alcohol during their lifetime, and the majority of the alcohol abstainers were female. More than half (55.5%) of the male participants were current drinkers compared to 12.8% of the females. Males also consumed alcohol more frequently than females. During a year, 38.6% of males consumed alcohol daily, and 82.7% of males had consumed alcohol within the past 30 days.

**Table 1:** Alcohol consumption pattern and behaviour

Characteristics	Total	Female	Male	
		N (%)	N (%)	*p*-value
Drinking behaviour (*n* = 2815)				< 0.001
Lifetime abstainer	1814 (64.4)	1495 (81.1)	319 (32.8)	
Former drinker	224 (8.0)	111 (6.1)	113 (11.6)	
Current drinker	777 (27.6)	236 (12.8)	541 (55.6)	
Consumed alcohol in past 12 months(*n* = 1001)				< 0.001
Consumed	777 (77.6)	236 (67.8)	541 (82.7)	
Not consumed	224 (22.4)	111 (32.2)	113 (17.3)	
Frequency in 12 months (*n* = 777)				< 0.001
Daily	259 (33.3)	50 (21.2)	209 (38.6)	
Three or four times a week	40 (5.2)	13 (5.5)	27 (5.0)	
Once or twice a week	126 (16.2)	37 (15.7)	89 (16.5)	
Four to seven times a month	118 (15.2)	30 (12.7)	88 (15.2)	
One to three times a month	234 (30.1)	106 (44.9)	128 (23.7)	
Consumed alcohol within past 30 days (n = 777)				< 0.001
Consumed	571 (73.5)	143 (60.6)	428 (82.7)	
Not consumed	206 (26.5)	93 (39.4)	113 (17.3)	
Occasions of at least one drink				
Mean ± Standard deviation	17.7 ± 0.5	15.9 ± 3.9	18.3 ± 0.6	
Average standard alcoholic drinks on one occasion				
Mean ± Standard deviation	7.7 ± 0.3	4.4 ± 0.3	8.8 ± 0.3	
Binge drinking (*n* = 571)				< 0.001
Yes	397 (69.5)	67 (46.9)	330 (77.1)	
No	174 (30.5)	76 (53.1)	98 (22.9)	

On average, drinking respondents consumed 7.7 ±0.3 standard alcoholic drinks on a single occasion. The average amount of standard alcoholic drinks was almost twice as high among males (8.8 ± 0.3) as among females (4.4 ± 0.3). Overall, nearly 70% of the respondents who consumed alcohol in the last 30 days were binge drinking relatively more among male (77.1%) than female (46.9%).

### Alcohol consumption during the past seven days

**Fig. 1:**
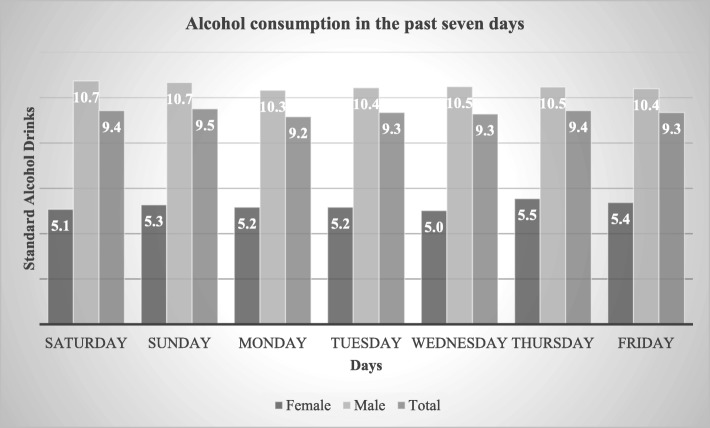
Alcohol consumption in the past seven days

Among 425 individuals (100 females and 325 males), who had consumed alcohol in the last 7 days, the average daily consumption was 9.4 standard alcohol drinks. One standard drink refers to 14 g of pure alcohol. The average consumption among males was almost twice as high as the average for females. The highest average standard alcohol drink consumption was observed during Sunday (9.5) and the lowest consumption during Monday (9.2) Fig 1.

### Association between lifetime alcohol consumption and sociodemographic variables

Gender, age, and ethnicity were found to be significantly associated with lifetime alcohol consumption (Table 2). Males had 16 (95% CI: 12.1–21.1) times higher odds of ever having consumed alcohol compared to females. Older adults of 45–65 years had 30% higher odds of ever having consumed alcohol than the younger adults of 24–44 years of age. Likewise, respondents belonging to disadvantaged ethnic communities had a 6.1 (95% CI, 4.9–6.3) times higher odds of ever having consumed alcohol than other ethnic groups.

**Table 2:** Association between lifetime alcohol consumption and sociodemographic variables

Characteristics	Unadjusted odds ratio (95% CI)	Adjusted odds ratio (95% CI)
Sex		
Female	1	1
Male	8.8 (7.4–10.5) *	16.0 (12.1–21.1) *
Age		
24–44 years	1	1
45–65 years	1.5 (1.2–1.7) *	1.3 (1.0–1.6) *
Educational level		
High education	1	1
Low education	1.2 (1.0–1.4)*	0.8 (0.6–1.0)
Marital Status		
Unmarried and divorced/widowed	1	1
Married	1.0 (0.8–1.3)	0.8 (0.6–1.1)
Ethnicity		
Advantaged ethnicity	1	1
Disadvantaged ethnicity	3.5 (3.0–4.1) *	6.1 (4.9–6.3) *
Occupational status		
Agriculture	1	1
Homemaker	0.5 (0.4–0.6) *	0.9 (0.7–1.1)
Labor	5.5 (3.5–8.6) *	1.3 (0.8–2.2)
Employee	1.6 (1.3–2.0) *	1.0 (0.8–1.4)
Unemployed	2.0 (1.6–2.6) *	0.6 (0.4–0.8) *

*CI*: Confidence Interval, *p < 0.05

### Association between binge drinking and sociodemographic variables

Binge drinking was also more prominent among males (Table 3). Males were 7.9 (95% CI: 4.3–14.6) times more likely to be binge drinking compared to females. Unlike lifetime alcohol consumption, educational and occupational status were associated with binge drinking behaviour. Lower educational status and being a homemaker, employed or unemployed were found to be protective to binge drinking compared to having a higher education or to be employed in agriculture.

**Table 3:** Association between binge drinking and sociodemographic variables

Characteristics	Unadjusted odds ratio (95% CI)	Adjusted odds ratio (95% CI)
Sex		
Female	1	1
Male	3.9 (2.6–5.8) *	7.9 (4.3–14.6) *
Age		
24–44 years	1	1
45–65 years	1.5 (1.1–2.2) *	1.4 (0.9–2.2)
Education		
High education	1.0	1
Low education	0.9 (0.6–1.3)	0.5 (0.3–0.9) *
Marital Status		
Unmarried and divorced/widowed	1	1
Married	2.0 (1.1–3.4) *	1.4 (0.7–2.5)
Ethnicity		
Advantaged ethnicity	1	
Disadvantaged ethnicity	1.0 (0.7–1.5)	1.6 (1.0–2.5)
Occupational status		
Agriculture	1	1
Homemaker	0.2 (0.1–0.4) *	0.5 (0.2–0.9) *
Labor	1.5 (0.7–3.2)	1.0 (0.4–2.2)
Employee	0.6 (0.3–0.9) *	0.4 (0.2–0.8) *
Unemployed	0.5 (0.3–0.8) *	0.3 (0.2–0.5) *

*CI*: Confidence Interval, *p < 0.05

## Discussion

Alcohol consumption is one of the modifiable risk factors associated with various non-communicable diseases, injuries and mental illness [20]. However, studies on prevalence, behavioural patterns of alcohol consumption and associated social correlates of binge drinking in Nepal are limited. Thus, as a part of the COBIN study, these elements have been studied in a semi-urban area of Pokhara Municipality.

Our study showed that the prevalence of lifetime alcohol abstainers (64.4%) in Western Nepal is high compared to the global average where almost half (48%) of the world population abstained from alcohol use [12]. This is less than in the South East Asia Region where more than three-quarters of the population has never consumed alcohol in their lifetime [12]. The observed prevalence of current drinking and binge drinking in the present study was much higher than reported by national level STEPS survey [17], which reported a current binge drinking behaviour of 28% among males and 7.1% among females. Similarly, the same study reported that among the current drinkers, 18.6% of males and 2.9% of females indulged in heavy episodic drinking. With respect to many western countries [21, 22], the current alcohol consumption behaviour is lower in western Nepal but resembles the pattern in many low and middle-income countries [12, 23, 24].

In accordance with many studies [25–27] conducted in developing countries, the current study also found that the consumption and frequency of alcohol consumption was higher among males than females in Western Nepal. This difference in gender on alcohol consumption behaviour can be linked to social and cultural norms which identify males drinking alcohol as normal behaviour while in case of females, drinking alcohol is considered an immoral act and is prohibited in various households [28]. In the context of Nepal, a significant proportion of females consume home-brewed alcohol rather than industrially produced alcohol [11]. However, along with the changing lifestyle, nature of work and entertainment purposes, society’s perception of alcohol consumption is also changing. Hence, the increasing trends of higher consumption of alcohol among females; however, this is more an urban phenomenon [28, 29]. This is also supported by one of the interesting findings of the study that shows increased use of alcohol for both males and females during the weekend. Nonetheless, it must be noted that though males (8.8 standard drink) drink almost twice the amount of alcohol compared to females (4.4 standard drink) in western Nepal, both genders met the criteria of HED (binge drinking), which is alarming.

Similar to other studies [30, 31], the likelihood of lifetime alcohol consumption is higher among older adults as compared to the young, this study also showed similar trend. However, studies reporting a decline in alcohol consumption with increasing age also exist [32, 33]. Though age was not significantly associated with binge drinking in this study, various studies have shown that alcohol misuse among young people is a growing public health issue.

In addition, different ethnicities reflect either positive or negative attitudes towards alcohol consumption behaviour. Usually, upper-caste such as Brahmin and minority ethnic groups such as Muslim have restrictive views on alcohol use [34], while some ethnicities in Nepal consider alcohol as pure offerings to God with significant religious importance. Alcohol consumption in these ethnicities is embedded in the lifestyle and reflects the long-term ethnicity acceptance of alcohol-related behaviour [11, 34]. A similar result is reflected in this study too, where disadvantaged ethnic communities, including Dalit and Janajati minorities were more than six times more likely to consume alcohol during their lifetime compared to people from upper-caste.

A study on correlates of adult binge drinking in a British cohort showed that being an unskilled worker, male and having a lower educational status was associated with high binge drinking [35]. This was not seen in the present study. Interestingly, those with a higher educational level were found to have a higher binge drinking behaviour than people with a low educational level. It can be speculated that, in many cases attaining higher education act as a stressor [36]. Evidence suggests that substance abuse occurs in the presence of a mental and physical stressor. Thus, in order to avoid such education-induced stress, people may binge drink. Concurrent to this evidence, a study done in the United States of America [37] showed that college students were drinking more and were suffering from clinically significant alcohol-related problems as compared to their non-college attending peers. Thus, the relationship between higher attainment of education and reduced alcohol use as reported by various studies [38, 39] may be rather complicated. However, the current study is unable to explain how and to what level education plays a role in forming drinking behaviour. This furthermore highlights the need for the further robust study.

Likewise, the present study found that people involved in agriculture tend to have higher odds of lifetime alcohol consumption and binge drinking compared to those who are either employed, unemployed or homemakers. This may be due to a high demand for physical labour in the agricultural field, which may act as a stressor. Nonetheless, evidence suggests that individual unemployment is one of the risk factors for alcohol abuse [40]. Hence, further study is required to establish a causal relationship between occupational status and alcohol consumption behaviour.

### Strengths and limitations

This study is one of the pioneer studies to report alcohol consumption patterns and significant socio-demographic correlates of Western Nepal. These findings might be helpful for policymakers and program planners in the context of ongoing efforts to reduce alcohol consumption, and most importantly addressing the morbidity and mortality due to heavy episodic drinking or binge drinking. However, the study has some limitations. The cross-sectional design of the study did not allow an explanation of the causality of alcohol consumption. The risk of recall bias, especially unintentional (information bias in alcohol consumption in the last 12 months), should be considered while interpreting the results of this study. Likewise, we cannot rule out the optimistic bias that occurs when a respondent tends to underreport the alcohol consumption behavior as heavy alcohol consumption, in general, is known as negative behaviour or over-report alcohol consumption as it is also seen as a sign of affluent living or trending culture.

However, in a resource-constrained settings like Nepal where evidence on behavior and pattern of alcohol consumption and its associated factors are almost non-present, this study provides an important testimony on the extent of alcohol consumption in Western Nepal. Thus, this study done at a semi-urban area of Pokhara Municipality which lies in Gandaki Province under the new federal structure of Nepal can provide essential relevant information in alcohol consumption behaviour for the region aiding to develop a tailored policy and program for the region. This study also can be a reliable base for further studies investigating alcohol consumption behavior in the country so that nationally tailored informed health promotion can be developed to address the public health problem of alcohol abuse.

## Conclusion

In conclusion, alcohol consumption behavior such as lifetime alcohol consumption, current drinking behaviour and binge drinking were predominantly higher among males compared to females in Western Nepal. The prevalence of binge drinking was reported to be about 70% among those who had consumed alcohol during the last 30 days. Likewise, males between 45 and 65 years of age from specific ethnicities (Dalit and Janjati) had a higher risk of lifetime alcohol consumption than other participants. Hence, this calls for tailored behaviour change information and intervention programs to reduce alcohol consumption at the community level.

## Data Availability

The data is available on request from COBIN study group Principle Investigator Dinesh Neupane (last author), who can be contacted through neupane.dinesh@gmail.com.

## Abbreviations

COBIN: Community Based Management of Non-communicable diseases in Nepal
DALYs: Disability Adjusted Life Years
FCHVs: Female Community Health Volunteers
HED: Heavy episodic alcohol drinking
SDGs: Sustainable Development Goals
WHO: World Health Organization
